# Enhancing EHR Interoperability and Security through Distributed Ledger Technology: A Review

**DOI:** 10.3390/healthcare12191967

**Published:** 2024-10-02

**Authors:** João Carlos Ferreira, Luís B. Elvas, Ricardo Correia, Miguel Mascarenhas

**Affiliations:** 1 Faculty of Logistics, Molde University College, NO-6410 Molde, Norway; joao.carlos.ferreira@iscte-iul.pt (J.C.F.); luis.elvas@iscte-iul.pt (L.B.E.); 2 Center for Research of Technologies and Architecture, Instituto Universitario de Lisboa ISCTE-IUL, ISTAR, 1649-026 Lisboa, Portugal; 3 INESC INOV-Lab, 1000-029 Lisbon, Portugal; 4 BioGHP, 1000-260 Lisboa, Portugal; ricardo@bioghp.com; 5 Department of Community Medicine, Information and Health Decision Sciences (MEDCIDS), Faculty of Medicine, University of Porto, 4200-319 Porto, Portugal

**Keywords:** electronic health records (EHRs), distributed ledger technology (DLT), health data interoperability, data security in healthcare, medical data integration

## Abstract

The management and exchange of electronic health records (EHRs) remain critical challenges in healthcare, with fragmented systems, varied standards, and security concerns hindering seamless interoperability. These challenges compromise patient care and operational efficiency. This paper proposes a novel solution to address these issues by leveraging distributed ledger technology (DLT), including blockchain, to enhance data security, integrity, and transparency in healthcare systems. The decentralized and immutable nature of DLT enables more efficient and secure information exchange across platforms, improving decision-making and coordination of care. This paper outlines a strategic implementation approach, detailing timelines, resource requirements, and stakeholder involvement while addressing crucial privacy and security concerns like encryption and access control. In addition, it explores standards and protocols necessary for achieving interoperability, offering case studies that demonstrate the framework’s effectiveness. This work contributes by introducing a DLT-based solution to the persistent issue of EHR interoperability, providing a novel pathway to secure and efficient health data exchanges. It also identifies the standards and protocols essential for integrating DLT with existing health information systems, thereby facilitating a smoother transition toward enhanced interoperability.

## 1. Introduction

The healthcare industry is responsible for generating immeasurable quantities of data on a daily basis, and according to RBC Capital Markets, in 2019, approximately 30% of the global data volume was generated by the healthcare sector [1]. In 2019, hospitals alone produced a staggering 50 petabytes of data per year, encompassing electronic health records (EHRs), medical images, laboratory tests, and sensor readings [2]. The importance of this topic is highlighted in this EU report about Implementing the European Health Data Space Across Europe [3]. Despite recent EU initiatives to advance AI in healthcare, adoption remains low due to slow implementation, lack of targeted policies, and trust issues. While progress has been made in health data regulation, AI usage is limited to specific areas and further financial and policy support is needed to promote broader adoption. The Commission aims to address challenges by improving investment, healthcare data exchange, and upskilling professionals, while fostering trust and translating research into clinical practice [4].

Despite the vast amounts of generated healthcare data, these data are characterized by being private, sensitive, and personal, presenting a barrier to accessibility. Compliance with data privacy regulations, such as the General Data Protection Regulation (GDPR) [5], is paramount.

In terms of data management, each hospital has its modus operandi for organizing and saving information about patients [6]. In some cases, there are even differences between services within the same hospital. Physicians generally value the flexibility and efficiency of unstructured text fields when writing patients’ clinical information [7].

One of the biggest challenges when exchanging EHRs is the difference between them, so it is necessary to create an exchange format to unlock the flow of health data across borders [8].

### 1.1. Importance of EHR Exchange and Interoperability

The healthcare industry is increasingly recognizing the critical importance of EHR exchange and interoperability [7]. These concepts refer to the seamless sharing and utilization of patient health information across different healthcare settings and systems. The integration and interoperability of EHR systems are pivotal for several reasons, each contributing to enhanced healthcare delivery, reduced errors, increased efficiency, and improved patient experience [8].

Interoperable EHR systems enable healthcare providers to access comprehensive patient histories, regardless of where the patient received previous care. This continuity of care is essential for making informed clinical decisions, reducing the risk of medical errors, and avoiding redundant tests and procedures. When healthcare providers have real-time access to a patient’s complete medical history, they can perform personalized medicine.

The efficient exchange of EHRs streamlines administrative processes and reduces healthcare providers’ time on manual data entry and retrieval. This efficiency translates into lower operational costs and allows healthcare professionals to focus more on patient care than administrative tasks. Additionally, interoperability minimizes duplicate testing and unnecessary procedures, reducing healthcare costs for providers and patients.

Interoperable EHR systems are invaluable for public health monitoring and medical research. Aggregated data from interoperable systems can be used to track disease outbreaks, monitor public health trends, and evaluate the effectiveness of treatments and interventions. This data-driven approach enhances the ability of public health officials and researchers to respond to health crises and develop evidence-based healthcare policies and practices.

From the patient’s perspective, safety is significantly bolstered through the exchange of EHRs. Interoperability ensures that critical information, such as allergies, medications, and past medical procedures, is readily available to all treating healthcare professionals. This reduces the likelihood of adverse drug interactions, allergic reactions, and other preventable medical errors. Moreover, interoperable systems facilitate timely alerts and reminders for necessary follow-ups and preventive care, further safeguarding patient health.

Interoperability empowers patients by giving them greater control over their health information. Patients can easily access their medical records, share them with healthcare providers, and stay informed about their health status. This transparency fosters patient engagement and encourages individuals to actively manage their health and wellness.

### 1.2. Overview of Distributed Ledger Technology (DLT) in Healthcare

Distributed ledger technology (DLT) is already a reality in healthcare. Several problems have led to the need to use blockchain technology in healthcare systems.

The first one is drug counterfeiting, which is a significant and growing issue in the healthcare industry, endangering patient safety and wasting income [9]. As previously mentioned, data privacy and security are of utmost importance, and it is critical to follow the GDPR. The lack of standardization in storing information is another crucial problem that can lead to delays in patient treatment if healthcare professionals do not receive the information needed at the right time [10].

Blockchain has the potential to solve all these problems [10]. Interoperability, as mentioned in the previous section, can play a significant role in improving healthcare systems. Data integrity can be maintained at all levels, preventing malicious actors from having access to and manipulating the data. Blockchain also uses data encryption, with only the receiver being able to decrypt the content using his keys, which promotes the biggest layer of security. Being a distributed decentralized technology, it also prevents maintenance costs in current healthcare systems.

This paper is organized into the following sections:

1. Introduction—outlines the problem and objectives of the study.
2. Literature review methodology—describes the approach taken to review the relevant literature.
3. Key results and discussion—presents the major findings from the literature review and their implications.
4. Interoperability of health information systems—discusses the current status of fast healthcare interoperability resources (FHIR) and its role in healthcare.
5. Challenges and solutions—addresses existing problems, implementation issues, and proposed solutions for improving interoperability.
6. Conclusion and future work—summarizes the findings and suggests directions for future research.

## 2. Literature Review

The healthcare sector is increasingly reliant on digital technologies for managing and exchanging patient information [11]. However, the interoperability of health data systems remains a significant challenge, impacting the quality and efficiency of patient care [12]. This systematic review aims to explore the current landscape of data interoperability in eHealth, with a particular focus on the potential of blockchain and distributed ledger technology (DLT) to address these challenges [13].

Interoperability in the healthcare sector is the ability of healthcare systems to share, interpret, and use electronic health records (EHRs) coherently.

Interoperability in healthcare refers to the ability of the healthcare system to communicate, exchange, interpret, and use electronic health records (EHRs) coherently [14]. The lack of interoperability can lead to fragmented care, medical errors, and increased healthcare costs [15]. As such, improving data interoperability is crucial for enhancing patient outcomes, streamlining healthcare processes, and enabling more effective population health management [16].

Recent technological advancements, particularly in blockchain and DLT, have shown promise in addressing interoperability issues [17]. These technologies offer potential solutions for secure, transparent, and efficient health data exchange. However, the implementation of such technologies in healthcare settings is still in its early stages, and their effectiveness and scalability remain areas of active research [18].

This review seeks to synthesize the current evidence on data interoperability in eHealth, with the following objectives:

1. To identify the key challenges and opportunities in health data interoperability.
2. To evaluate the potential of blockchain and DLT in enhancing data exchange within eHealth systems.
3. To assess the current state of implementation and effectiveness of interoperability solutions.
4. To highlight gaps in the existing literature and suggest directions for future research.

By analyzing the latest research in this field, this review aims to provide valuable insights for healthcare professionals, policymakers, and researchers working towards improving healthcare data interoperability. The findings of this review may inform future strategies for developing more integrated, efficient, and patient-centered healthcare information systems.

### 2.1. Methods and Inclusion Criteria

For the development of this review, it was implemented the Preferred Reporting Items for Systematic Reviews and Meta-Analysis (PRISMA) methodology [19]. This methodology was designed to help authors transparently exhibit the background, methods, and results of their systematic review [19]. As it provides a rigorous set of guidelines for reporting, it also assures that the results can be recreated.

In the context of this review, this methodological rigor is crucial for drawing robust conclusions, identifying research gaps, and providing actionable insights for policymakers and practitioners aiming to improve health data exchange and interoperability.

### 2.2. Search Strategy

To develop the search strategy, we targeted keywords that aligned with the review’s objectives. These keywords were carefully selected to capture the intersection of data interoperability, electronic health record (EHR) exchange, eHealth standards, and emerging technologies such as blockchain and distributed ledger technology (DLT). The keywords were then organized into the following search query to obtain the articles used in this review:

(“Data Interoperability” OR “EHR Exchange”) AND “eHealth” AND “Standards” AND (“Blockchain” OR “DLT”)

This search query was designed to capture studies that address the challenges and opportunities in health data interoperability, with a specific focus on the potential role of blockchain and DLT in enhancing data exchange within eHealth systems.

The search was conducted on Scopus [20], a comprehensive abstract and citation database, in July 2024, using this same search query. Scopus was chosen for its broad coverage of peer-reviewed literature across various disciplines, including healthcare informatics and technology. The following table contains the selected keywords, organized into sections (concepts, context, application, and technology), and the number of articles obtained through them. There were also some limitations to the search, focusing on peer-to-peer articles and reviews written in English.

This section presents the main findings from our review of data interoperability in eHealth. We begin by outlining the search and selection process using the PRISMA flow diagram, followed by a summary of the key findings.

**Figure 1:**
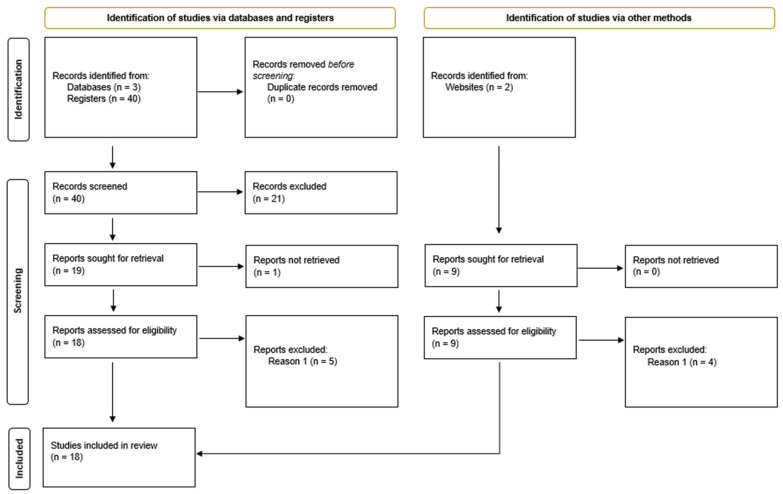
PRISMA flow diagram.

Our systematic search strategy yielded a total of 37 initial records, as it was shown in Table 1. As the search was only conducted on Scopus, there were no duplicated articles. Additionally, we included an article that was identified independently due to its relevance and contribution to the topic. Figure 1 presents the PRISMA flow diagram, illustrating the step-by-step process of the study selection.

### 2.3. Results

The screening process involved a systematic, multi-step approach to ensure a thorough and unbiased selection of relevant studies. Initially, we conducted a comprehensive scan of titles and abstracts to make a preliminary selection, eliminating articles that did not focus on the most important aspect of our review, data interoperability, or EHR exchange. In this step, 21 articles were excluded.

Subsequently, we excluded articles that could not be retrieved in full text, ensuring that our analysis would be based on complete information. From the previous articles, only one could not be retrieved.

The final step involved an in-depth analysis of the remaining articles, carefully evaluating their content against our criteria to make the final selection. The selection criteria required that each article focus on at least three of the four keyword sections identified in our search strategy. In this final step, eight articles did not meet the inclusion criteria. This rigorous process allowed us to identify the most pertinent and high-quality studies for our review while minimizing the risk of overlooking relevant research.

All the articles selected were published between 2018 and 2023, with the year 2023 yielding the most studies. This trend may be explained by the growing recognition of the potential for blockchain and distributed ledger technologies to address long-standing challenges in healthcare data interoperability. The surge in research interest likely reflects the maturation of these technologies, coupled with an increasing emphasis on secure, efficient health data exchange in the wake of global health crises and the rapid digitalization of healthcare services. Additionally, the evolution of eHealth standards and the pressing need for robust, interoperable systems to support remote care and telemedicine may have further fueled research in this area.

**Figure 2:**
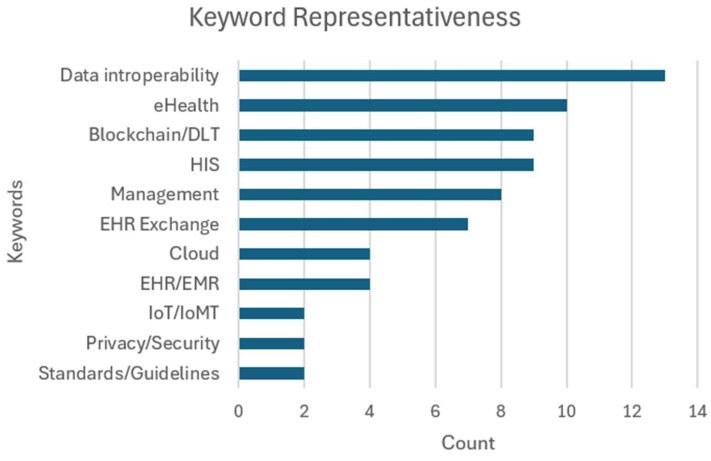
Keyword representativeness.

In Figure 2, as expected, “Data interoperability” emerges as the most prevalent theme, appearing in 86.7% of the reviewed articles. “eHealth” follows closely, present in 10 articles, out of the total 15. It is also relevant to mention that “Blockchain/DLT” and Health Information Systems (“HIS”) are equally represented in 60% of the articles each, indicating a strong interest in exploring blockchain technologies within the context of existing health information systems. Table 2 attributes each paper to their respective identified keywords.

## 3. Literature Review Results Discussion

### 3.1. Outcome Analysis and Principal Findings

In this sub-chapter, we dive into the analysis of outcomes from the papers included in this literature review. The reviewed articles collectively highlight the significant steps developed in enhancing interoperability within healthcare systems, particularly through the integration of blockchain technology, cloud computing, and IoT/IoMT solutions. By examining these studies, we aim to identify the common findings, conclusions, and future research directions that can pave the way for more robust and efficient healthcare information systems.

Several studies propose blockchain-based frameworks to improve healthcare data interoperability and security. Reference [21] demonstrates practical solutions for protecting healthcare data and supporting efficient data sharing. Similarly, other research efforts present unified health information system frameworks that integrate blockchain with IoT, big data analytics, and artificial intelligence to facilitate real-time data sharing and develop smart strategies for addressing healthcare challenges [22].

A common theme across some studies is the use of standardized data formats, particularly the Health Level 7 Fast Healthcare Interoperability Resources (HL7 FHIR), to ensure consistency and ease of data exchange. One study developed a smart-contract authentication-assisted HL7-FHIR framework for an interoperable e-healthcare solution [30], while another proposed an FHIR Practice Guide (PG) to facilitate stakeholders’ understanding and implementation of FHIR standards [26].

Researchers also explored novel approaches to semantic interoperability. The SemBox system [29] achieved a maximum classification accuracy of 85.71% in enabling collaboration between heterogeneous health monitoring wearable devices. Another study proposed smart-fuzzy-linked rules for the cross-domain (Smart-FCD) framework [31] to ensure interoperability across multiple platforms and systems.

Studies also focused on enhancing security and privacy in EHR systems. Reference [23] proposed an innovative solution leveraging blockchain’s decentralized nature combined with advanced encryption techniques. Another study [24] constructed a PHR blockchain architecture for international health record exchange, which has been deployed in Southeast Asian countries.

A medical data interoperability through collaboration (MeDIC) framework [32] improved upon cloud-based IoMT by utilizing translation resources at the network edge, reducing uplink traffic, and improving response time for real-time medical applications. Another study [33] outlined a blockchain-based platform for storing and managing electronic medical records in a cloud environment.

However, challenges remain in implementing these solutions at scale. Some proposed frameworks, such as the blockchain-based framework for interoperable electronic health records (BCIF-EHR) [25], are still in the prototype or testing stages. There is a lack of standardized implementation methods for blockchain-based health information systems, as highlighted in the survey on blockchain-based self-sovereignty and patient data records [34]. Additionally, the trade-offs between decentralization, privacy/security, and system performance need careful consideration when designing blockchain-based healthcare systems.

The review on developing infrastructure for cross-border health data exchange [27] identified several challenges, including the lack of proper infrastructure, legislative and administrative issues, and the underestimation of semantic interoperability problems. Another study [28] found that while certain security measures can reduce technical interoperability problems, some privacy regulations may increase semantic and organizational interoperability issues.

Reference [14] addressed key aspects of EHR interoperability and blockchain technology. This study found that document-based data stores effectively model EHR structures for efficient storage, search, and retrieval. It also highlighted the challenge of interoperability across different blockchain platforms for inter-organization health data exchange. By reviewing data interoperability solutions across various blockchain engines, this work provides valuable insights into achieving the objectives of EHR exchange while maintaining privacy and security.

These findings collectively demonstrate the potential of blockchain and related technologies in improving healthcare interoperability and data management, while also highlighting the complexities and challenges that need to be addressed for successful real-world implementation.

### 3.2. Discussion

Future work prospects for both the original research articles and review studies emphasize the continued development and testing of blockchain-based frameworks in real-world healthcare settings. For instance, the patient-centric healthcare framework [21] and the blockchain-based framework for interoperable EHR [25] call for implementation in actual electronic health record systems to evaluate their effectiveness and scalability. Similarly, the PHR blockchain architecture [24] aims to extend its application for precision medicine across different countries.

In terms of reviews, future research directions include developing standardized implementation methods for blockchain in healthcare, integrating centralized and decentralized systems, and exploring advanced privacy-preservation techniques such as on-chain/off-chain data partitioning. The reviews also highlight the need for government policies to promote the adoption of interoperability standards like FHIR and the importance of technical expertise and infrastructure to support large-scale implementations.

### 3.3. Conclusions

In conclusion, while blockchain and DLT show significant potential in addressing long-standing challenges in healthcare data interoperability, further research and development are necessary to overcome existing barriers and realize the full benefits of these technologies in practical healthcare settings. The field’s rapid evolution suggests that continued innovation and collaboration among researchers, healthcare providers, and policymakers will be crucial in shaping the future of interoperable, secure, and efficient healthcare information systems.

## 4. Interoperability of Health Information Systems

This chapter discusses Fast Healthcare Interoperability Resources (FHIR), a global industry standard for exchanging healthcare data electronically [35].

### 4.1. Standards and Protocols for Interoperability

Standards are crucial for data exchange. Without them, exchanging data and achieving integration at the local, regional, national, or international level would be almost impossible [36]. As such, there are several healthcare standards: the Internacional Classification of Diseases (ICD) [37], FHIR, SNOMED clinical terms [38], and current procedural terminology [39]. The FHIR standard stands out due to its simplicity and flexibility, combining the best features of previous data exchange standards into a common specification [40].

The FHIR standard is a critical standard developed by Health Level Seven International (HL7), which facilitates the interoperability of health information systems [41]. It defines a set of “resources” representing various clinical and administrative data elements, ensuring consistent data formats and structures across systems. By utilizing modern web technologies such as RESTful APIs, XML, and JSON, FHIR simplifies the implementation and integration of interoperable solutions, enabling seamless communication and data exchange between disparate health information systems [42].

### 4.2. Integration with Existent Health Information Systems

The FHIR design allows for smooth integration with existing health information systems without requiring extensive overhauls. Its modular resource-based architecture enables it to be layered over current standards and technologies, providing compatibility with legacy systems. By employing standardized APIs and data formats, FHIR facilitates real-time access to patient information, enhancing clinical workflows and enabling healthcare providers to retrieve and share critical data efficiently. This integration capability supports the continuity of care and improves overall system functionality.

### 4.3. Data Sharing and Exchange Processes

The FHIR standard significantly improves data sharing and exchange processes by offering a standardized framework that promotes interoperability. Its use of consistent data models and resources allows different health systems to exchange information seamlessly, reducing barriers caused by incompatible formats. FHIR’s support for RESTful APIs enables efficient, secure, and scalable data exchanges, ensuring healthcare providers have timely access to accurate patient data. This enhances care coordination, decision-making, and patient outcomes by enabling a more connected and responsive healthcare ecosystem.

### 4.4. Case Studies or Pilot Implementations

The Geisinger and the Steele Institute for Health Innovation had the goal of optimizing Geisinger’s Family Care Application to facilitate communication between caregivers and care teams. The approach followed involved converting its application programming interface from a standard EHR web service to HL7 FHIR. It resulted in improved access to patient information, including primary healthcare partner contacts, care partner names, appointments, provider notes, and medication details [43].

With Humana and MongoDB, the objective was to enhance data exchange for Humana’s members and providers. They implemented FHIR APIs to enable the seamless sharing of health data, leading to improved interoperability, data accuracy, and patient engagement [44].

At the University of Washington, researchers leveraged FHIR for data sharing across research systems, which led to streamlined data access, improved collaboration, and accelerated research [45].

Finally, at Ochsner Health the aim was to improve patient care through data exchange. They implemented FHIR for seamless data sharing across Ochsner’s health system and the results showed enhanced care coordination, data accessibility, and patient outcomes [46]. Table 3 presents a summary of FHIR cases.

## 5. Challenges and Solutions

The healthcare industry has been struggling with data silos, where patient data are scattered across different entities and are often inaccessible when needed [47]. Blockchain technology has emerged as a promising solution to address this challenge, as it can serve as a trusted decentralized database for health information exchange. Blockchain-enabled decentralization can help minimize the vendor lock-in issue that has plagued the healthcare industry and provide a secure and transparent platform for data sharing.

One of the key challenges in implementing blockchain for healthcare interoperability is the issue of data transaction volume. The high volume of clinical data generated in healthcare can be a significant bottleneck for blockchain-based systems. Additionally, concerns around patient privacy and security are critical, as healthcare data are highly sensitive. To address these challenges, researchers have proposed mechanisms such as digital access rules, data aggregation, and data immutability, which can facilitate the transition from institution-centric to patient-centric data sharing.

Another challenge is patient engagement, as the success of blockchain-enabled interoperability relies heavily on patients’ willingness to participate and share their data. Incentives for patients to actively engage with and contribute to the blockchain-based system are crucial [48].

Despite these challenges, the potential benefits of blockchain technology in healthcare interoperability are significant. Blockchain can enable data liquidity, allowing patients to have more control over their health data and facilitate seamless data sharing across different healthcare providers [48]. As the healthcare industry continues to evolve towards a more patient-centric model, blockchain-based solutions can play a crucial role in breaking down data silos and improving overall interoperability.

Table 4 summarizes the challenges and impacts of different aspects of distributed ledger technology (DLT) in healthcare.

The key challenges facing DLT adoption in healthcare, as highlighted in this table, reflect the complexities inherent in integrating advanced technologies into critical sectors like healthcare. Scalability and interoperability issues remain among the top barriers, as healthcare systems must handle large volumes of sensitive data while ensuring seamless interaction between legacy systems and new DLT platforms.

Data privacy and security are paramount concerns due to stringent regulations like GDPR and HIPAA, and the need to strike a balance between transparency and confidentiality. Failure to address these privacy concerns would not only violate regulations but also damage trust, a crucial factor in the healthcare sector.

Technical complexity, integration hurdles, and stakeholder resistance also present significant challenges. Healthcare organizations need to invest in the technical expertise required for DLT implementation and manage the cultural shift necessary to foster widespread adoption. Stakeholder education and early-stage pilot projects are necessary to alleviate concerns and demonstrate the value of DLT in improving operational efficiency, enhancing patient care, and ensuring regulatory compliance.

Ultimately, addressing these challenges through a combination of technical innovations, regulatory adaptations, and stakeholder engagement will be essential for DLT’s success in healthcare.

Table 5 represents the proposed solutions and mitigation strategies for various challenges in enhancing scalability, privacy, security, and more, in the implementation of DLT in healthcare.

Table 6 summarizes integration with emerging technologies and future trends in DLT for healthcare.

The challenge of semantic interoperability also stands out, with solutions like the SemBox system enabling collaboration across different health monitoring devices [49].

Other frameworks, such as the smart-fuzzy cross-domain (Smart-FCD) system, highlight how cross-platform interoperability can be achieved across various healthcare systems [50].

Security and privacy in EHR systems remain a critical concern. Blockchain’s decentralized nature combined with encryption techniques presents promising solutions, as demonstrated in [51], which proposed a blockchain architecture for managing international health records. However, these solutions face challenges, such as scalability, the trade-offs between decentralization, security, and system performance, and the lack of standardized implementation methods.

A notable limitation identified across the studies is that many blockchain-based frameworks are still in the prototype stage, requiring further testing and real-world validation. Challenges such as infrastructure gaps, legislative issues, and privacy regulations that complicate interoperability have also been identified, particularly in cross-border health data exchanges [52].

In conclusion, while blockchain and related technologies offer promising pathways to overcoming interoperability challenges, their widespread adoption will depend on overcoming practical implementation barriers. Future research should focus on addressing these limitations by testing blockchain frameworks in real-world settings, developing standardized methods for implementation, and refining techniques for privacy preservation. Additionally, support from governmental bodies and the creation of robust infrastructures are essential to enabling large-scale interoperability in healthcare systems.

## 6. The Integral Role of Blockchain in Enhancing Estonia’s Digital Healthcare Ecosystem

Estonia’s healthcare system is widely regarded as one of the most advanced in the world, largely due to its comprehensive use of digital technology [53]. The country’s implementation of a nationwide electronic health record system in 2008 has been a key driver of this digital transformation, enabling seamless sharing of medical records and patient data across hospitals, clinics, and other healthcare providers [54].

Blockchain technology plays a crucial role in further enhancing the security, privacy, and transparency of Estonia’s digital healthcare infrastructure. Specifically, the government utilizes a blockchain-based solution called KSI Blockchain to safeguard the integrity of sensitive healthcare data.

Data integrity: Blockchain’s immutable nature ensures that once data are recorded, they cannot be altered or tampered with, thereby preserving the integrity of medical records [55,56]. This is of critical importance in the healthcare industry, where trust and accurate record-keeping are essential for effective patient care.

Security: Blockchain technology provides an additional layer of security by decentralizing the storage of patient data and cryptographically linking records. This ensures that health data are protected from breaches or unauthorized access, as any attempt to tamper with the records would be immediately detectable.

Transparency and auditing: Blockchain allows patients and healthcare providers to see a complete audit trail of who accessed or modified data, ensuring transparency and empowering patients with control over their personal information. This helps to build trust in the healthcare system [53,54,55,56,57].

Decentralization: Unlike traditional centralized healthcare databases, blockchain’s decentralized architecture minimizes the vendor lock-in issue that has plagued the industry. This enables more efficient sharing of data and reduces the risk of data silos, ensuring that crucial information is accessible when needed.

The integration of blockchain technology has been instrumental in enhancing the security, transparency, and overall efficiency of Estonia’s digital healthcare ecosystem.

## 7. Conclusions

The interoperability of health information systems is crucial for advancing the quality and efficiency of healthcare delivery. The ability of different systems and platforms to exchange and utilize information effectively offers significant benefits for both healthcare professionals and patients. The main benefits are identified in Table 7.

In summary, interoperability is a key component in building a more efficient, secure, and patient-centered healthcare system. While technical and regulatory challenges are significant, the potential benefits for care quality, efficiency, and innovation justify the continued investment in solutions that promote integration and collaboration across different health information systems. The significance of EHR interoperability for seamless healthcare services is discussed in this study with regard to EHR ownership, structure, and sharing. An extensive study of massive data stores for EHR modeling, open standards for EHR interpretation, and healthcare ontologies for EHR knowledge representation is presented in this research. This facilitates the analysis of how different approaches to the problems of non-uniform semantics and the diverse EHR structure for semantic interoperability have been taken by scholars.

The interoperability assurances of the two widely used open standards for EHR were assessed. In order to reflect the heterogeneous EHR structure, a comparison study of data storage is conducted utilizing consistency semantics and performance measurements. It is shown beyond doubt that document-based data stores are highly capable of modeling the EHR structure in terms of efficient storage, retrieval, and search.

The protection of patient privacy is ranked as a critical component of interoperable solutions in this assessment.

By categorizing the literary works on privacy preservation for EHR into well-known strategies, the review gives a thorough analysis of the literature. In order to evaluate their implementation in the suggested framework for achieving patient-centric EHR ownership, the blockchain-based privacy preservation mechanisms were also examined.

The study highlights the importance of implementing blockchain technology in EHRs since it provides a viable solution for EHR traceability, immutable EHR storage, and IAM with pseudo-anonymity. But in light of the difficulties in using blockchain technology for EHR administration, our analysis confirms previous conclusions, namely that on-chain data requires a lot of processing power and is susceptible to privacy breaches when using decentralized ledgers.

The poll assesses the possibility of using blockchain to provide trustworthy, interoperable, and privacy-preserving solutions for data transfers amongst various stakeholders in the healthcare industry for patient-centric services. The structure of the prototype MyBlockEHR for trust-based, privacy-preserving EHR administration is a significant contribution of our work. Its use in conjunction with smart contracts and on- and off-chain data partitioning reveals that it outperforms centralized EHR systems and non-partitioned blockchain-based EHR management frameworks in terms of performance, access control, and verifiability.

This paper demonstrates how another research problem for the interorganizational exchange of health reports with interoperability goals in healthcare data is interoperability across blockchain platforms. To achieve the goals of EHR sharing, data interoperability solutions across various blockchain engines are examined. Our analysis reveals the need for high-quality cross-chain data-sharing studies in the healthcare domain. Therefore, further prototype testing and integration techniques for trust-based healthcare services are encouraged by our study of healthcare data interoperability.

### 7.1. Future Work

Future research in the following areas is necessary, as shown by the comprehensive survey’s findings and the proposed framework including experimental results.

In EHRs, semantic interoperability—particularly through ontologies—is shown to be quite limited in terms of use cases and applications via study, exhibiting a restricted range. These ontologies are also directed by RDF and OWL structures, which concentrate on representations. They may be used in conjunction with deep learning techniques and natural language processing to provide more accurate and better interpretation.

Privacy preservation techniques—used for maintaining privacy—are being explored. Blockchain-based privacy preservation strategies are stimulating more research into data partitioning strategies for on- and off-chain storage.

The next frontier in blockchain-based EHR management research will be patient-centric privacy protection. This will stimulate more research into data flow monitoring, patient-controlled access management, and data provenance tracking.

Blockchain-integrated systems may be used to create data-sharing incentives that will motivate patients to voluntarily provide more information for study in the future.

### 7.2. Blockchain Frameworks for EHR Interoperability

Current blockchain systems are based on EHR structures and bespoke semantics. Adopting open standards will enhance the semantic interoperability of the EHR framework. Issues with high power usage and the lack of scalability plague blockchain technologies. Big data issues, like data volume and velocity, affect EHR administration. More research is required to determine how to minimize the on-chain storage and on-chain calculations of EHRs. The privacy of patient data is at risk due to the distributed ledger in blockchain, which distributes data in a distributed system. Future studies will focus on developing blockchain-based solutions that make use of privacy protection strategies. In Healthcare 4.0, it is critical to establish smart contracts and access control strategies for patient-controlled audits. Future research will focus on developing verifiable, tamper-resistant off-chain storage.

Cross-chain interoperability: Healthcare data should be seamlessly exchanged between systems through cross-chain interoperability. The focus is on the seamless transmission of data across various blockchain-based platforms. Healthcare data exchanges must prioritize non-intermediated solutions and protect patient privacy. Creating a framework for exchanging data between two blockchain-based healthcare ecosystems while protecting patient privacy is the most important scientific problem this study poses.

### 7.3. Digital Wallets for Data Exchange in Blockchain

In the era of digital transformation, the emergence of blockchain technology has revolutionized the way we approach data exchange and management. Digital wallets have become a crucial component in this ecosystem, serving as a secure and convenient platform for users to store, transact, and control their digital assets [48,58,59].

Cryptocurrency wallets are the primary means by which individuals can interact with blockchain networks [60]. These wallets provide customers with the ability to send and receive virtual currency or tokens, as well as monitor their balance through direct interaction with blockchains [61]. Digital wallets can be categorized into three main types: software, hardware, and paper wallets. Software wallets are further divided into web, mobile, and desktop applications, each with its own set of features and security measures.

The significance of digital wallets in the blockchain ecosystem extends beyond just the management of cryptocurrencies.

The architecture of a digital wallet. The basic structure of a digital wallet consists of a unique identifier, typically a private/public key pair, which allows users to securely access and manage their digital assets. When users interact with blockchain-based applications, their digital wallets are used to authorize transactions and invoke corresponding smart contracts.

The integration of asymmetric cryptographic algorithms in digital wallets enhances the security and privacy of online transactions.

The widespread adoption of mobile technology has also contributed to the growth of digital wallets. More than 120 mobile money projects have been deployed in around 70 emerging markets, demonstrating the potential for digital wallets to revolutionize financial services and payment systems [48].

The success of digital wallets in the blockchain ecosystem is not without its challenges. While mobile payments are becoming increasingly common, the adoption of digital wallets for financial transactions remains limited in some regions. We are currently engaged in a project to implement this and this document serves as the starting point.

### 7.4. Enhancing Data Privacy and Compliance in Decentralized Ledger Technology Frameworks

As decentralized ledger technology continues to gain prominence across various industries, the need to address data privacy and regulatory compliance concerns has become crucial. While the inherent security features of distributed ledgers offer significant benefits, the decentralized nature of these systems can pose challenges in aligning with data privacy regulations, such as the General Data Protection Regulation.

To address this, advanced encryption techniques and privacy-preserving methods can play a vital role in limiting access to sensitive data while still leveraging the security advantages of decentralized ledger technology. Zero-knowledge proofs, for instance, can enable parties to verify the validity of a transaction without revealing the underlying data, while homomorphic encryption allows for data processing without the need for decryption. Additionally, off-chain storage solutions or permissioned blockchains can provide a means to store and manage sensitive information in a more controlled and privacy-preserving manner [60].

Furthermore, access control mechanisms, such as identity-based encryption and multi-signature authentication, can ensure that only authorized parties can view or modify specific data, reducing the risk of breaches. These measures, along with regular audits and transparent governance models, can help align decentralized ledger technology frameworks with GDPR and other privacy regulations [61].

The design of privacy-friendly data repositories for decentralized ledger technology has been a subject of growing research interest. Practitioners need to complement the technical framework with rigorous privacy risk management, including conducting privacy risk analyses, identifying strategies to address such risks, and preparing privacy impact assessments to enhance accountability and transparency. Blockchain technology has the potential to provide businesses with a means to nurture trust with customers regarding collected data, but this requires addressing the compatibility challenges between the decentralized approach of blockchain and the data subject rights enshrined in regulations like the GDPR [61].

Aligning technical innovation with regulatory requirements and healthcare needs involves strategies for scalable and compliant DLT solutions in EHR interoperability.

The healthcare industry has long grappled with achieving seamless interoperability between disparate electronic health record systems. Fragmented data sources, legacy infrastructure, and a highly regulated environment have hindered the efficient exchange and integration of critical healthcare information [62]. However, the rise of transformative technologies, such as distributed ledger technology, offers a promising avenue to address these longstanding barriers and foster cross-sector collaboration.

By leveraging the unique properties of blockchain, including decentralization, immutability, and enhanced security, healthcare organizations can develop scalable and compliant solutions that align technical innovation with regulatory requirements and practical healthcare needs [63]. The blockchain’s ability to serve as a trusted, decentralized database can help mitigate the issue of data silos, enabling the seamless exchange of patient records across multiple entities within the healthcare ecosystem. Furthermore, the technology’s inherent transparency and auditability can aid in addressing regulatory concerns, such as data privacy and security, while maintaining the integrity and traceability of medical information [63].

Existing research has highlighted the potential of blockchain-based electronic health record systems to enhance interoperability, security, and cost-effectiveness. By fostering cross-sector collaboration, stakeholders can identify strategies that leverage the unique capabilities of blockchain to develop solutions that are not only technologically advanced but also adhere to the stringent regulatory requirements of the healthcare industry.

This collaborative approach can help ensure that the development of DLT-based solutions for EHR interoperability is aligned with the practical needs of healthcare providers, patients, and regulators.

## Figures and Tables

**Table 1:** Keyword selection process.

Concepts	Context	Application	Technology	Limitations
Data interoperability EHR exchange	eHealth	Standards	Blockchain DLT	Document type: review, article Source Type: journal Language: English
5865 documents				
425 documents				
284 documents				
52 documents				
37 documents				

**Table 2:** Papers by keyword.

Keyword	Articles	Count	%
Data interoperability	[14,21,22,23,24,25,26,27,28,29,30,31,32]	13	86.7%
eHealth	[21,22,23,24,25,26,29,30,33,34]	10	66.7%
EHR/EMR	[14,21,23,24,25,28,30,33,34]	9	60%
Blockchain/DLT	[14,21,22,23,24,25,33,34]	9	60%
Privacy/Security	[14,23,24,25,27,28,33,34]	8	53.3%
Management	[14,23,24,25,26,29,32]	7	46.7%
EHR exchange	[14,23,24,27,30,33,34]	7	46.7%
IoT/IoMT	[21,29,31,32]	4	26.7%
Cloud	[21,22,32,33]	4	26.7%
Standards/guidelines	[26,27]	2	13.3%
HIS	[22,34]	2	13.3%

**Table 3:** Summary of case studies using FHIR.

Implementation	Goal	Approach	Results
Geisinger and the Steele for Health Innovation	Optimize Geisinger’s family care application for caregiver communication	Convert API from standard EHR web services to HL7 FHIR services	Improved access to patient information, including contacts, appointments, provider notes, and medication details
Humana and MongoDB	Enhance data exchange for Humana’s members and providers	Implement FHIR APIs for seamless health data sharing	Improved interoperability, data accuracy, and patient engagement
University of Washington	Share data across research systems	Leverage FHIR for streamlined data access and collaboration	Accelerated research and improved data sharing
Ochsner Health	Improve patient care through data exchange	Implement FHIR for seamless data sharing	Enhanced care coordination, data accessibility, and patient outcomes

**Table 4:** The key challenges DLT faces in healthcare and the impacts on operational efficiency, patient care, regulatory compliance, and stakeholder adoption.

Category	Challenges	Impact
Scalability issues	- Performance and scalability problems as transaction and data volumes increase. - Decentralized nature leads to slower transaction speeds and higher latency.	- Timely data access is crucial for patient care; delays can impede medical decisions, affecting patient outcomes. - Increased transaction costs may strain healthcare budgets, limiting the long-term viability of DLT implementations.
Data privacy and security	- Balancing transparency of DLT systems with strict healthcare data privacy regulations (e.g., GDPR, HIPAA). - Ensuring security while maintaining transaction visibility across the network.	- Unauthorized access or data breaches could compromise patient confidentiality and result in regulatory violations. - Failure to address privacy concerns could lead to legal repercussions, financial penalties, and loss of trust among users.
Interoperability with existing systems	- Integrating DLT solutions with legacy health information systems (HIS) and other disparate platforms. - Lack of standardization across healthcare platforms, leading to incompatibility.	- Inefficient data sharing between healthcare providers could result in redundant tests, delayed care, and fragmented services. - Poor interoperability affects care coordination and reduces the overall efficiency of healthcare delivery.
Data consistency and integrity	- Ensuring consistent and synchronized data across distributed nodes is challenging. - Immutable blockchain records complicate error correction processes.	- Inconsistent or corrupt data can result in incorrect clinical decisions, endangering patient safety. - Implementing robust consensus mechanisms and audits is necessary to ensure data accuracy and integrity.
Regulatory and compliance issues	- Navigating healthcare-specific regulations and DLT-related policies can be complex and uncertain. - Existing healthcare regulations may not fully align with the decentralized characteristics of DLT.	- Delays in DLT adoption due to uncertainty in how regulations apply, potentially leading to non-compliance. - Legal uncertainties may hinder large-scale deployment of DLT in healthcare.
Technical complexity and integration	- DLT systems involve high technical complexity, including cryptography, consensus mechanisms, and network protocols. - Specialized skills required for development, implementation, and management of DLT systems.	- Higher costs and longer implementation timelines may deter adoption by healthcare organizations. - Healthcare providers may require training or external partnerships, increasing dependency on technology providers.
Stakeholder resistance and adoption	- Resistance from healthcare professionals, administrators, and patients due to unfamiliarity with DLT or concerns about workflow disruption. - Fear of change in clinical processes or management.	- Lack of stakeholder buy-in could prevent successful adoption, leading to ineffective utilization of the technology. - Early engagement through education, pilot projects, and hands-on training can help mitigate resistance and promote adoption.

**Table 5:** Summarizes the proposed solutions and mitigation strategies for tackling key challenges in implementing distributed ledger technology in healthcare settings.

Challenge	Proposed Solution	Mitigation Strategy
Enhancing scalability	Implement layer-2 solutions such as state channels or sidechains to improve scalability. Use optimized consensus algorithms to increase transaction throughput.	Regularly assess and upgrade the network infrastructure to handle increasing data volumes and transaction loads.
Strengthening data privacy and security	Employ advanced encryption methods, zero-knowledge proofs, and privacy-preserving technologies to secure health data on the blockchain.	Ensure compliance with data protection regulations by incorporating privacy-by-design principles in the development process.
Facilitating interoperability	Develop and adopt standardized interfaces and protocols for DLT integration with existing HIS. Utilize interoperability frameworks and middleware solutions.	Collaborate with standard-setting organizations and industry groups to align DLT solutions with existing healthcare standards.
Ensuring data consistency and integrity	Implement robust consensus mechanisms and data validation processes to ensure data consistency across distributed ledgers.	Regularly audit and verify data integrity through automated tools and periodic reviews.
Addressing regulatory and compliance issues	Engage with regulatory bodies to shape and adapt regulations for DLT in healthcare. Develop compliance frameworks tailored to DLT technology.	Ensure thorough legal and regulatory reviews during the development and implementation phases to align with existing laws.
Managing technical complexity	Invest in training and development for technical staff to build expertise in DLT. Use user-friendly tools and platforms to simplify implementation.	Partner with technology providers and consultants who specialize in DLT to support implementation and maintenance.
Overcoming stakeholder resistance	Conduct educational programs and workshops to demonstrate the benefits of DLT. Engage stakeholders early in the development process to gather input and address concerns.	Pilot DLT solutions in smaller settings to showcase their effectiveness and build confidence among users.

**Table 6:** Highlights the trends shaping the future of DLT in healthcare and provides actionable considerations to help organizations integrate these technologies, adapt to evolving standards, and enhance their DLT strategies.

Category	Trend	Consideration
Integration with emerging technologies	DLT is increasingly being integrated with AI, IoT, and big data analytics to enhance capabilities in healthcare.	Explore opportunities to leverage AI, IoT, and big data analytics to create more comprehensive solutions for health data management.
Evolution of standards and regulations	The regulatory landscape for DLT in healthcare is expected to evolve with new standards and guidelines emerging.	Stay updated on regulatory changes and actively participate in shaping industry standards to ensure both compliance and innovation.
Adoption of hybrid blockchain models	Hybrid blockchain models that combine public and private blockchains are gaining traction for their flexibility and efficiency.	Evaluate the benefits of hybrid models for your specific use case and consider their implementation in your DLT strategy.
Enhanced focus on interoperability	There will be a growing emphasis on improving interoperability between DLT systems and other health information systems.	Invest in developing and adopting standards and protocols that facilitate seamless data exchange and integration.
Increased emphasis on patient consent and control	Patient empowerment and control over their own health data will become the central focus in DLT implementations.	Implement features that allow patients to manage their consent and access to data, ensuring transparency and trust.
Scalability improvements	Ongoing advancements in blockchain technology will continue to address scalability challenges.	Stay informed on technological developments and adopt innovations that enhance the scalability and performance of DLT systems.
Real-world applications and case studies	Increasing real-world applications and case studies will provide valuable insights into the practical use of DLT in healthcare.	Learn from successful implementations and case studies to guide your own DLT projects and strategies.

**Table 7:** Highlights how interoperability in healthcare improves patient care, decision-making, and efficiency, while also emphasizing the importance of addressing data security and privacy challenges.

Aspect	Benefit/Challenge	Description
Improved care coordination	Benefit	Interoperability facilitates the smooth exchange of information between various providers and services, ensuring access to complete and up-to-date patient data. This enhances coordination, and continuity of care, reduces duplication of tests, and minimizes medical errors.
Support for decision-making	Benefit	Interoperable data enables healthcare professionals to make more informed, evidence-based decisions. It provides a fuller view of the patient’s health status, leading to more accurate diagnoses and effective treatment plans.
Operational efficiency	Benefit	Interoperable systems reduce manual data entry and rework, improving operational efficiency and lowering administrative costs. Automation and process integration lead to better resource management and enhance the overall patient experience.
Data security and privacy	Challenge	Interoperability presents challenges related to data security and privacy. Systems must have robust data protection measures and comply with regulations like GDPR and HIPAA to safeguard patient information against unauthorized access and data breaches.
Facilitation of innovation and research	Benefit	Effective data integration supports innovation and research by giving access to large volumes of interoperable data. Researchers can conduct detailed analyses, identify new trends and patterns, and contribute to advancements in disease treatment and prevention.

## Data Availability

No data are available. No new data were created or analyzed in this study.

## References

[1] RBC Capital Markets|Navigating the Changing Face of Healthcare Episode. https://www.rbccm.com/en/gib/healthcare/story.page.

[2] 4 Ways Data Is Improving Healthcare. World Economic Forum. https://www.weforum.org/agenda/2019/12/four-ways-data-is-improving-healthcare/.

[3] General Data Protection Regulation (GDPR)—Legal Text. General Data Protection Regulation (GDPR). https://gdpr-info.eu/.

[4] Patients at Risk Because NHS Hospitals Using Different Record-Keeping Systems|Imperial News|Imperial College London. Imperial News. https://www.imperial.ac.uk/news/194269/patients-risk-because-nhs-hospitals-using/.

[5] Rosenbloom S.T., Denny J.C., Xu H., Lorenzi N., Stead W.W., Johnson K.B. (2011). Data from Clinical Notes: A Perspective on the Tension between Structure and Flexible Documentation. J. Am. Med. Inform. Assoc..

[6] Exchange of Electronic Health Records across the EU|Shaping Europe’s Digital Future. https://digital-strategy.ec.europa.eu/en/policies/electronic-health-records.

[7] Turbow S., Hollberg J.R., Ali M.K. (2021). Electronic Health Record Interoperability: How Did We Get Here and How Do We Move Forward?. JAMA Health Forum.

[8] EHR Interoperability: Benefits & Best Practices. https://www.elationhealth.com/resources/blogs/ehr-interoperability-and-primary-care.

[9] Blackstone E.A., Fuhr J.P., Pociask S. (2014). The Health and Economic Effects of Counterfeit Drugs. Am. Health Drug Benefits.

[10] 101 Blockchains, Blockchain for Healthcare: Use Cases and Applications. 101 Blockchains. https://101blockchains.com/blockchain-for-healthcare/.

[11] Kruse C.S., Stein A., Thomas H., Kaur H. (2018). The use of Electronic Health Records to Support Population Health: A Systematic Review of the Literature. J. Med. Syst..

[12] Lehne M., Sass J., Essenwanger A., Schepers J., Thun S. (2019). Why digital medicine depends on interoperability. npj Digit. Med..

[13] Agbo C.C., Mahmoud Q.H., Eklund J.M. (2019). Blockchain Technology in Healthcare: A Systematic Review. Healthcare.

[14] Sonkamble R.G., Phansalkar S.P., Potdar V.M., Bongale A.M. (2021). Survey of Interoperability in Electronic Health Records Management and Proposed Blockchain Based Framework: MyBlockEHR. IEEE Access.

[15] Menachemi N., Collum T.H. (2011). Benefits and drawbacks of electronic health record systems. Risk Manag. Health Policy.

[16] McKibbon K.A., Lokker C., Handler S.M., Dolovich L.R., Holbrook A.M., O’Reilly D., Tamblyn R., Hemens B.J., Basu R., Troyan S. (2012). The effectiveness of integrated health information technologies across the phases of medication management: A systematic review of randomized controlled trials. J. Am. Med. Inform. Assoc..

[17] Hasselgren A., Kralevska K., Gligoroski D., Pedersen S.A., Faxvaag A. (2020). Blockchain in healthcare and health sciences—A scoping review. Int. J. Med. Inform..

[18] McGhin T., Choo K.-K.R., Liu C.Z., He D. (2019). Blockchain in healthcare applications: Research challenges and opportunities. J. Netw. Comput. Appl..

[19] PRISMA Statement. https://www.prisma-statement.org/.

[20] Scopus—Document Search|Signed in. https://www.scopus.com/search/form.uri?display=basic#basic.

[21] Gohar A.N., Abdelmawgoud S.A., Farhan M.S. (2022). A Patient-Centric Healthcare Framework Reference Architecture for Better Semantic Interoperability Based on Blockchain, Cloud, and IoT. IEEE Access.

[22] He W., Zhang J.Z., Wu H., Li W., Shetty S. (2022). A Unified Health Information System Framework for Connecting Data, People, Devices, and Systems. J. Glob. Inf. Manag..

[23] Benaich R., El Mendili S., Gahi Y. (2023). Advancing Healthcare Security: A Cutting-Edge Zero-Trust Blockchain Solution for Protecting Electronic Health Records. HighTech Innov. J..

[24] Lee H.-A., Kung H.-H., Udayasankaran J.G., Kijsanayotin B., Marcelo A.B., Chao L.R., Hsu C.-Y. (2020). An architecture and management platform for blockchain-based personal health record exchange: Development and usability study. J. Med. Internet Res..

[25] Reegu F.A., Abas H., Gulzar Y., Xin Q., Alwan A.A., Jabbari A., Sonkamble R.G., Dziyauddin R.A. (2023). Blockchain-Based Framework for Interoperable Electronic Health Records for an Improved Healthcare System. Sustainability.

[26] Nan J., Xu L.-Q. (2023). Designing Interoperable Health Care Services Based on Fast Healthcare Interoperability Resources: Literature Review. JMIR Med. Inform..

[27] Natsiavas P., Mazzeo G., Faiella G., Campegiani P., Dumortier J., Stan O., Nalin M., Martinez D.M., Theodouli A., Moschou K. (2021). Developing an infrastructure for secure patient summary exchange in the EU context: Lessons learned from the KONFIDO project. Health Inform. J..

[28] Shrivastava U., Song J., Han B.T., Dietzman D. (2021). Do data security measures, privacy regulations, and communication standards impact the interoperability of patient health information? A cross-country investigation. Int. J. Med. Inform..

[29] Pathak N., Mukherjee A., Misra S. (2023). SemBox: Semantic Interoperability in a Box for Wearable e-Health Devices. IEEE J. Biomed. Heal. Inform..

[30] Sreejith R., Senthil S. (2023). Smart Contract Authentication assisted GraphMap-Based HL7 FHIR architecture for interoperable e-healthcare system. Heliyon.

[31] Anitha K., Balasubramani M., S V.P.K. (2023). Smart-fcd: Iot data interoperability using sensor based fuzzy linked rules for cross domain applications. Malays. J. Comput. Sci..

[32] Jaleel A., Mahmood T., Hassan M.A., Bano G., Khurshid S.K. (2020). Towards Medical Data Interoperability Through Collaboration of Healthcare Devices. IEEE Access.

[33] Kaur H., Alam M.A., Jameel R., Mourya A.K., Chang V. (2018). A Proposed Solution and Future Direction for Blockchain-Based Heterogeneous Medicare Data in Cloud Environment. J. Med. Syst..

[34] Houtan B., Hafid A.S., Makrakis D. (2020). A Survey on Blockchain-Based Self-Sovereign Patient Identity in Healthcare. IEEE Access.

[35] Vorisek C.N., Lehne M., Klopfenstein S.A.I., Mayer P.J., Bartschke A., Haese T., Thun S. (2022). Fast Healthcare Interoperability Resources (FHIR) for Interoperability in Health Research: Systematic Review. JMIR Public Health Surveill..

[36] Imler T.D., Vreeman D.J., Kannry J., Finnell J.T., Dixon B.E. (2016). Healthcare Data Standards and Exchange. Clinical Informatics Study Guide: Text and Review.

[37] ICD-10–CM International Classification of Diseases, Tenth Revision, Clinical Modification (ICD-10-CM). https://www.cdc.gov/nchs/icd/icd-10-cm/index.html.

[38] Stearns M.Q., Price C., Spackman K.A., Wang A.Y. (2001). SNOMED clinical terms: Overview of the development process and project status. Proc. AMIA Symp..

[39] A Hirsch J., Leslie-Mazwi T.M., Nicola G.N., Barr R.M., A Bello J., Donovan W.D., Tu R., Alson M.D., Manchikanti L. (2014). Current procedural terminology; a primer. J. NeuroInterv. Surg..

[40] Shah R. FHIR in Healthcare: A Comprehensive Guide. Osplabs. https://www.osplabs.com/insights/fhir-in-healthcare-guide/.

[41] What Is FHIR Fact Sheet.pdf. https://www.healthit.gov/sites/default/files/page/2021-04/What%20Is%20FHIR%20Fact%20Sheet.pdf.

[42] Weistra W. FHIR Is Transforming Interoperability in Healthcare—But What Exactly Is It?. Firely..

[43] 20-HL7-003 FCA Case Study _Web.pdf. https://www.hl7.org/documentcenter/public/case-studies/20-HL7-003%20FCA%20Case%20study%20_Web.pdf.

[44] 22HL7003 Case Study—MongoDB Humana.pdf. https://www.hl7.org/documentcenter/public/case-studies/22HL7003%20Case%20Study%20-%20MongoDB%20Humana.pdf.

[45] 23HL7007_CaseStudy-UniversityofWashington.pdf. https://www.hl7.org/documentcenter/public/case-studies/23HL7007_CaseStudy-UniversityofWashington.pdf.

[46] 21HL7002 Pfizer eSource Case Study-REV-Web.pdf. https://www.hl7.org/documentcenter/public/case-studies/21HL7002%20Pfizer%20eSource%20Case%20Study-REV-Web.pdf.

[47] Elvas L.B., Serrã C., Ferreira J.C. (2023). Sharing Health Information Using a Blockchain. Healthcare.

[48] Katuwal G.J., Pandey S., Hennessey M., Lamichhane B. (2018). Applications of Blockchain in Healthcare: Current Landscape & Challenges. arXiv.

[49] Gordon W.J., Agrawal A. (2018). Blockchain Technology for Healthcare: Facilitating the Transition to Patient-Driven Interoperability. Comput. Struct. Biotechnol. J..

[50] Daniel C., Ouagne D., Sadou E., Paris N., Hussain S., Jaulent M.-C., Kalra D. (2016). Cross border semantic interoperability for learning health systems: The EHR4CR semantic resources and services. Learn. Health Syst..

[51] Kasyapa M.S.B., Vanmathi C. (2024). Blockchain integration in healthcare: A comprehensive investigation of use cases, performance issues, and mitigation strategies. Front. Digit. Health.

[52] Duan K., Pang G., Lin Y. (2023). Exploring the current status and future opportunities of blockchain technology adoption and application in supply chain management. J. Digit. Econ..

[53] Nanda A. (2019). Consumers’ Intention to use Mobile Wallets. AIMS Int. J. Manag..

[54] Ullah H.S., Aslam S., Arjomand N. (2020). Blockchain in Healthcare and Medicine: A Contemporary Research of Applications, Challenges, and Future Perspectives. arXiv.

[55] Mertz L. (2018). (Block) Chain Reaction: A Blockchain Revolution Sweeps into Health Care, Offering the Possibility for a Much-Needed Data Solution. IEEE Pulse.

[56] Mackey T.K., Miyachi K., Fung D., Qian S., Short J.E. (2020). Combating Health Care Fraud and Abuse: Conceptualization and Prototyping Study of a Blockchain Antifraud Framework. J. Med. Internet Res..

[57] Khushalani J., Chandwani S., Shaikh A.S., Talreja B., Hande R. (2020). Blockchain: The Novel Way to Secure Confidence!. Proceedings of the International Conference on Electronics and Sustainable Communication Systems (ICESC).

[58] Shicheng W., Lin H., Gan W., Chen J., Yu P. (2023). The Next Internet Revolution. arXiv.

[59] Omodunbi T., Akindutire A.S., Awoyelu T.M., Ikono R., Gambo I. (2023). Integrating Asymmetric Cryptographic Digital Wallet for Online Services in Nigeria. Int. J. Inf. Eng. Electron. Bus..

[60] Suratkar S., Shirole M., Bhirud S. Cryptocurrency Wallet: A Review. Proceedings of the 2020 4th International Conference on Computer, Communication and Signal Processing (ICCCSP).

[61] Glew R., Tröger RSchmittner S.E. (2022). A solution approach for the anonymous sharing of sensitive supply chain traceability data. arXiv.

[62] Al-Abdullah M., Alsmadi I., AlAbdullah R., Farkas B. (2020). Designing privacy-friendly data repositories: A framework for a blockchain that follows the GDPR. Digit. Policy Regul. Gov..

[63] Kouroubali A., Katehakis D.G. (2022). Policy and Strategy for Interoperability of Digital Health in Europe. MEDINFO 2021: One World, One Health–Global Partnership for Digital Innovation.

